# Voltage-dependent anion channel (VDAC) is involved in apoptosis of cell lines carrying the mitochondrial DNA mutation

**DOI:** 10.1186/1471-2350-10-114

**Published:** 2009-11-09

**Authors:** Liu Yuqi, Gao Lei, Li Yang, Li Zongbin, Xu Hua, Wang Lin, Chen Rui, Liu Mohan, Wen Yi, Guan Minxin, Wang Shiwen

**Affiliations:** 1 Institute of Geriatric Cardiology, Chinese PLA General Hospital, Beijing, 100853, PR China; 2 Military Medical Science Academy of the Chinese PLA, Beijing, 100850, PR China; 3 Cincinnati Children's Hospital Medical Center, Division of Human Genetics, Cincinnati, OH 45229, USA

## Abstract

**Background:**

The mitochondrial voltage-dependent anion channel (VDAC) is increasingly implicated in the control of apoptosis. We have studied the effects the mitochondrial DNA (mtDNA) tRNA^Ile ^mutation on VDAC expression, localization, and apoptosis.

**Methods:**

Lymphoblastoid cell lines were derived from 3 symptomatic and 1 asymptomatic members of a family with hypertension associated with the A4263G tRNA^Ile ^mutation as well as from control subjects. Mitochondrial potential (ΔΨ_m_) and apoptosis were measured by flow cytometry; co-localization of VDAC and Bax was evaluated by confocal microscopy.

**Results:**

Expression of VDAC and Bax in mtDNA cell lines was found to be increased compared to controls, while expression of the small conductance calcium-dependant potassium channel (sK_Ca_) was unchanged. Confocal imaging revealed co-localization of VDAC/Bax on the outer mitochondrial membrane of A4263G cell lines but not from controls. Flow cytometry indicated that the mitochondrial potential was decreased by 32% in mutated cells versus controls while rates of apoptosis were increased (*P *< 0.05). The difference was attenuated by Cyclosporin A (CsA, 2 μM), a blocker of VDAC.

**Conclusion:**

We conclude that increased expression of mitochondrial VDAC and subcellular co-localization of VDAC/Bax increases mitochondrial permeability and apoptosis in cell lines carrying the mtDNA tRNA^Ile ^A4263G mutation.

## Background

Hypertension is an established risk factor for coronary heart disease, stroke, congestive heart failure and renal dysfunction, and is the major modifiable risk factor of poor prognosis in a variety of cardiovascular diseases. Multiple environmental and genetic factors are known to predispose to essential hypertension, with genetic predisposition contributing to 30-60% of the pathoetiology of the disease [1]. We previously reported on families with an inherited disposition to essential hypertension; some families showed an obvious pattern of maternal inheritance indicative of a mitochondrial disorder [2-5]. In a previous study on a large Chinese Han family with maternally-inherited hypertension we uncovered a mutation in the mitochondrial A4263G tRNA^Ile ^gene. The mutation was found to affect a nucleotide conserved from bacteria to human. Importantly, the change was inferred to influence amino acid charging of tRNA [6] and was therefore likely to lead to translational amino-acid substitutions in some mitochondrial proteins, with major consequences for mitochondrial function.

Recent studies have reported that the outer mitochondrial membrane voltage-dependent anion channel (VDAC) is associated with type 2 diabetes mellitus [7-9], an important finding in view of the link between diabetes and mitochondrial function [10,11]. There are 3 kinds of VDAC 1 to 3, but in human *VDAC1 *is highly expressed, which controls the transit of adenine nucleotides, Ca^2+^, and other metabolites [12,13] both into and out of the mitochondrion. The channel is also a constituent of the mitochondrial permeability transition pore (PTP) [14-16] and therefore is likely to play a central role in the control of apoptosis. To study the link between the mitochondrial tRNA^Ile ^A4263G mutation, high blood pressure, and apoptosis we established lymphoblastoid cell lines from individuals carrying the A4263G mutation and from controls. We report that the A4263G mutation is associated with changes in VDAC expression, localization, and levels of apoptosis.

## Methods

### Cell lines and culture conditions

**Figure 1:**
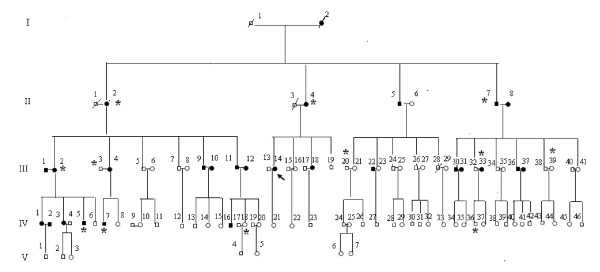
**The Han Chinese pedigree with a maternal pattern of inheritance of hypertension**. Affected individuals are indicated by filled symbols. The arrowhead denotes the proband. * denotes cardiac hypertrophy.

Informed consent, blood samples were obtained from all of the participating family members, under protocols approved by the ethics committee of Chinese PLA General Hospital and the Cincinnati Children's Hospital Medical Center Institute Review Board. Lymphoblastoid cell lines were immortalized by transformation with Epstein-Barr virus as described elsewhere [17]. Cell lines derived from 4 members of the Chinese family with a maternally-inherited predisposition to hypertension. Three individuals [II-4, III-14, III-18] displayed clinical hypertension, one individual [III-19] was asymptomatic (see Figure 1). Cell lines were also isolated by the same method from 3 genetically unrelated control individuals (A1, A2, A3). Cells were grown in RPMI 1640 medium (Gibco) supplemented with 15% fetal bovine serum (FBS).

### Expression of VDAC, Bax and sK_Ca_

Cells lines carrying the mtDNA tRNA^Ile ^4263 A→G and control cell lines were washed with ice-cold PBS and total RNA was isolated using the TRIzol reagent (Invitrogen) according to the manufacturer's instructions. RNA (2 μg) was treated with ribonuclease-free deoxyribonuclease and cDNA was synthesized using Moloney murine leukemia virus reverse transcriptase (Invitrogen Life Technologies, Carlsbad, CA); cDNA (2 μl) was subjected to 44 cycles of PCR amplification, generating a single specific amplification product of the expected size. PCR conditions were as follows: denaturation for 30 sec at 94°C, annealing for 1 min at 55°C (VDAC, Bax) or 58°C (sK_Ca_), and 45 sec extension at 72°C. PCR primers used in this study were: VDAC sense, 5'-CTG AGT ACG GCC TGA CGT TT-3'; antisense 5'-ACT CTG TCC CGT CAT TCA CA-3'; Bax sense 5'-GCA GCT TAA CGC ACC AAT TA-3', antisense 5'-CAG TTG AAG TTG CCG TCA GA-3'; sK_Ca _sense 5'-GCA GCT TAA CGC ACC AAT TA-3', antisense 5'-TGA GGG AAA GGA CCA CTG AT-3'. Glyceraldehyde 3-phosphate dehydrogenase (GAPDH) was used as the PCR internal control using primers 5'-CTG CAC CAC CAA CTG CTT AG-3' (sense) and 5'-TTC AGC TCA GGG ATG ACC TT-3' (antisense). PCR reactions were in the linear range and were performed in triplicate; products were visualized by electrophoresis on 1.5% agarose gels and stained with ethidium bromide. Band intensities were normalized to GAPDH amplified in parallel and means were calculated from the triplicate reactions.

For the study of Cyclosporin A (CsA, an inhibitor of VDAC) on expression levels of VDAC, Bax and sK_Ca_, cell lines were incubated in the presence of CsA (Novartis, 2 μM) for 16 h before further analysis.

### Confocal microscopy

Dual immunostaining was used to asssess the co-localization of Bax and VDAC-1 polypeptides. Cells were grown on glass coverslips in 6-well plates. After fixation (4% paraformaldehyde in PBS), specimens were blocked with 5% BSA for 15 min, and incubated overnight with 200 μl of primary antibody (anti-VDAC polyclonal antibody; Cell Signaling Technology, Danvers, MA; or anti-Bax polyclonal Antibody, NeoMarkers, Fremont, CA) diluted 1:100, followed by FITC green or rhodamine 123 labelled secondary antibody for 60 min at 37°C, respectively. After washing, slides were mounted with cover slips and imaged using a confocal laser scanning system (RADIANCE 2100, Bio-Rad, Hercules, CA). Excitation-emission used an Argon 488 nm laser in conjunction with a 505-525 nm filter for the Alexa Fluor 488, and a HeNe 543 laser with a 610 nm filter for the Alexa Fluor 546 [18].

### Mitochondrial membrane potential and apoptosis measurements

The mitochondrial membrane potential (ΔΨ_m_) was monitored using the fluorescent reporter probe JC-1. Lymphoblastoid cell lines were incubated with 0.1 μM JC-1 (Alexis; Portland, OR) for 10 min at 37°C [19]. After this loading period the cells were rinsed with phosphate-buffered saline (PBS)/bovine serum albumin and resuspended into 0.1 μM JC-1 in PBS/bovine serum albumin at room temperature [20]. ΔΨ_m _was measured by cytofluorimetry (FL3). To evaluate the effects of CsA on ΔΨ_m_, cell lines were pre-incubated with CsA (Novartis, 2 μM) for 30 min and washed 5× in PBS before measurements. For determinations of levels of apoptosis, cells were stained with Annexin V/FITC and PI stain and imaged using laser-scanning confocal microscopy (LSCM).

### Statistical analyses

All data were presented as  ± SD. Comparison of continuous variables was performed using the unpaired Student's *t *test. Statistical significance was set at *P *< 0.05. Statistical analysis used SPSS software (version 11.0; SPSS Inc, Chicago, IL).

## Results

### Expression of VDAC, Bax and sK_Ca_

**Figure 2:**
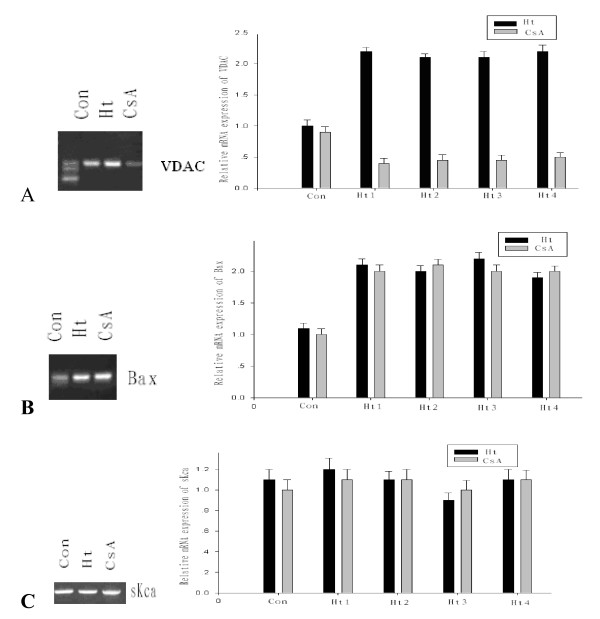
**Expression of VDAC, Bax and sK_Ca _mRNAs**. Con, cell line from the control group; Ht_1_, cell line from III-14; Ht_2_, cell line from II-4; Ht_3_, cell line from III-18; Ht_4_, cell line from III-19; CsA, cell line from III-14 after incubation with CsA (2 μM) for 16 h. A, expression of VDAC mRNA; B, expression of Bax mRNA; C, expression of sK_Ca _channel mRNA.

To study the effects of the mitochondrial tRNA^Ile ^A4263G mutation on mitochondrial function, lymphoblastoid cell lines were isolated from familial carriers of the mutation (Figure 1) and from controls. Quantitative RT-PCR was used to measure the mRNA expression levels of VDAC, Bax and sK_Ca_. As shown in Figure 2, the A4263G mutation was associated with a significant (*P *< 0.05) increase in the levels of expression of both VDAC and Bax. There was no change in the expression level of a control channel, sK_Ca_.

We then investigated the effects of Cyclosporin A (CsA), a selective inhibitor of VDAC, on VDAC and Bax mRNA levels. After incubation with CsA for 16 h, VDAC mRNA levels decreased significantly and even lower than the control levels (*P *< 0.05) while there was no significant change in the levels of either Bax or sK_Ca _mRNA (Figure 2).

### Co-localization of VDAC and Bax protein

**Figure 3:**
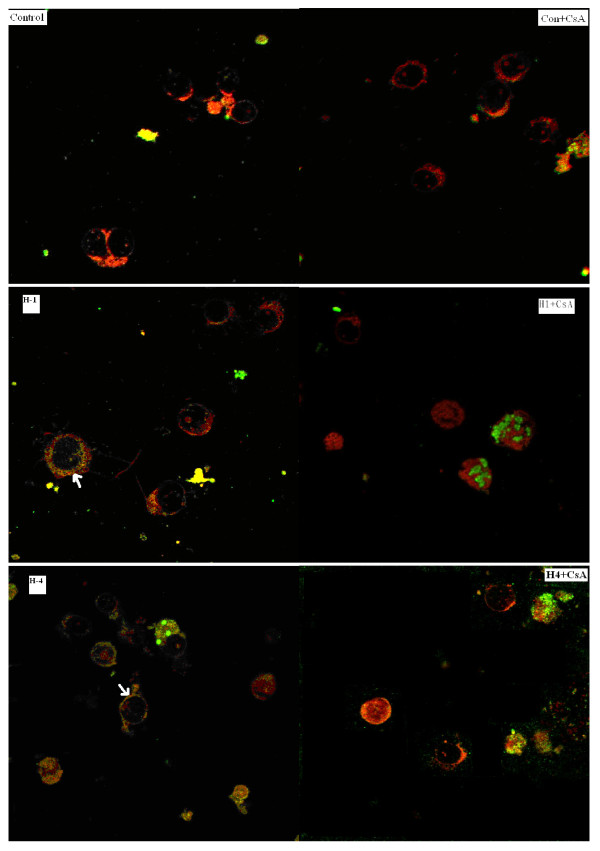
**Localization of mitochondrial VDAC and Bax by confocal microscopy**. Control, cell line from control group; Con+CsA, cell line incubated with CsA (2 μM) for 30 min; H-1, cell line from III-14; H-1+CsA, cell line incubated with CsA (2 μM) for 30 min; H-4, cell line from III-19; H-4+CsA, cell line incubated with CsA (2 μM) for 30 min. VDAC was stained with FITC-green (green fluorescence); Bax was stained with rhodamine 123 (red fluorescence).

VDAC was localized on the outer membrane of the mitochondrial, but Bax was expressed in the cytoplasm, transferred to outer membrane of mitochondrial and combined to VDAC under pathological state. To address the relative localizations of VDAC and Bax in cell lines carrying the mtDNA tRNA^Ile ^A4263G mutation, specific antibodies against these proteins were used for dual immunofluorescence on mutant cells and controls. Localization was imaged under confocal microscopy. Representative sections are shown in Figure 3.

Separate VDAC (green) and Bax (red) fluorescence was detected in control cell lines, indicating that the respective polypeptides were separately localized. In contrast, in cell lines from 2 tRNA^Ile ^A4263G subjects (III-14 and III-19) co-localization of VDAC and Bax was revealed by intense yellow fluorescence.

We also investigated the effects of CsA on co-localization. Cell lines were incubated with CsA for 30 min prior to analysis. Confocal imaging revealed that CsA treatment abolished co-localization of VDAC and Bax.

### Mitochondrial membrane potential (ΔΨ_m_) and apoptosis

**Figure 4:**
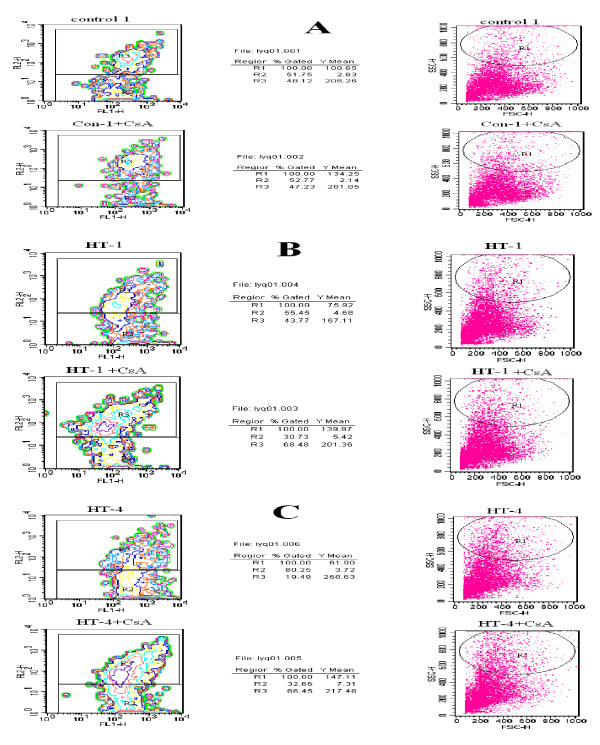
**Mitochondrial potentials (ΔΨ_m_) of cell lines from III-14, III-19 and from controls analyzed by flow cytometry**. The left and right figures show ΔΨ_m _detected by flow cytometry before and after incubation with CsA (2 μM) for 30 min, respectively. A, cell lines of the control group; B, III-14; C, III-19.

The magnitude of ΔΨ_m _is controlled by the activity of the mitochondrial K_ATP _(mitoK_ATP_) and mitoK_Ca _channels, as well as by the mitochondrial permeability transition pore (PTP) [21]. Cytometry images of lymphocytes loaded with JC-1, a specific reporter of ΔΨ_m_, revealed that membrane potential in tRNA^Ile ^A4263G cells was decreased by 32% compared to controls (*P *< 0.05). After incubation with CsA for 30 min the ΔΨ_m _of both control and the mutated cell lines was increased; the increase was 33.6% in control cells but was 84.4% in III-14 mutant cells and 137.7% in III-19 mutant cells (Figure 4).

**Figure 5:**
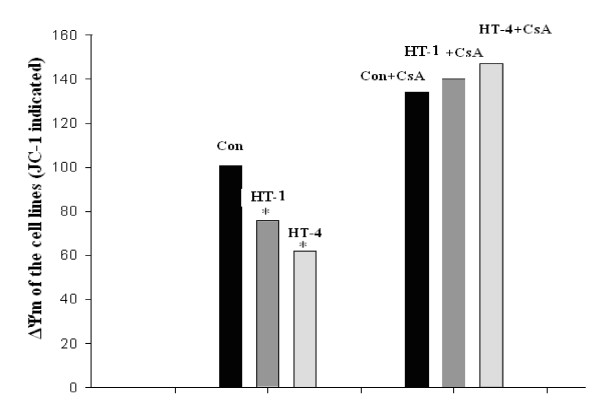
**Comparison of ΔΨ_m _of cell lines from patients and controls**. The average ΔΨ_m _value of cell lines from patients harboring the tRNA^Ile ^A4263G mutation was decreased by 32% compared to control cell lines. ΔΨ_m _values of cell lines from both controls and patients were improved by CsA; control values increased by 33.6%, III-14 values increased by 84.4%, and III-19 values increased by 137.7%.

We then compared ΔΨ_m _values between cell lines from controls and subjects carrying the A4263G mutation, both prior to and following incubation with CsA. As shown in Figure 5, ΔΨ_m _values were significantly decreased (*P *< 0.05) in both cell lines harboring the mutation. CsA pretreatment increased ΔΨ_m _in all cell lines, and after CsA treatment there was no significant difference between ΔΨ_m _values in control and mutant cells (Figure 5).

**Figure 6:**
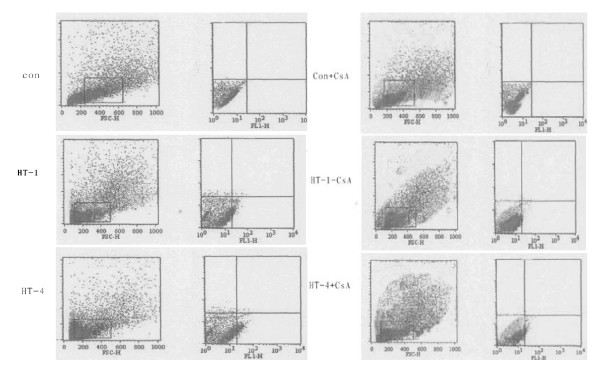
**Apoptosis of cell lines from III-14, III-19, and controls**. The left and right panels show flow cytometric analysis of apoptosis before and after incubation with CsA (2 μM) for 30 min, respectively.

To determine whether changes in VDAC mRNA levels, localization, and ΔΨ_m _values in lines from subjects carrying the A4263G mutation are associated with changes in apoptosis, cells were analyzed using Annexin V-FITC and flow cytometry, a sensitive assay for apoptosis. Levels of apoptosis were increased by 30% in cell lines carrying the mutation (Figure 6, left panel). However, the difference from controls was largely abolished by treatment with CsA; this reduced levels of apoptosis by 24.6% in control cells but by 56.9% in III-14 cells and by 67.1% in III-19 cells (Figure 6, right panel).

## Discussion

Hypertension is a major risk factor for cardiovascular disease. Approximately 1 billion individuals worldwide and 130 million in China suffer from hypertension, and the rates of morbidity and mortality associated with essential hypertension (EHT) continue to rise [22]. Epidemiological studies have indicated that the genetic variance underlying the predisposition to EHT ranges from 30-60% [1]. Mitochondrial DNA (mtDNA) mutations characterized by maternal inheritance may make a significant contribution [23-26]. The Framingham heart study [27] on blood pressure in 6421 participants from 1593 families estimated that the heritability due to maternal effects was 5% for multivariable-adjusted long-term average systolic blood pressure, while the heritability of diastolic blood pressure due to maternal effects was 4%.

We previously reported on a large Chinese Han family with a predisposition to hypertension that demonstrated a typical maternal pattern of inheritance. mtDNA sequence analysis revealed a A4263G mutation in the mtDNA tRNA^Ile ^gene that is extraordinarily conserved from bacteria to human. The voltage-dependent anion channel (VDAC) is a highly conserved protein located on the outer mitochondrial membrane [11]. VDAC, in association with ANT (adenine nucleotide translocator), mediates the transport of ATP and ADP both into and out of the mitochondrion [11]. VDAC closure inhibits the release of ATP from the mitochondrion and promotes the opening of K_ATP _channels in the plasma membrane. VDAC also mediates mitochondrial Ca^2+ ^and may play a key role in intracellular Ca^2+ ^signaling [28]. *t*Bid, a pro-apoptotic member of the Bcl_2 _family, closes VDAC, and this may partly account for the inhibition of VDAC reported during apoptosis [29]. In contrast, anti-apoptotic Bcl-XL prevents VDAC closure, a finding consistent with the interpretation that VDAC opening is anti-apoptotic [30]. VDAC also appears to be an anchor point for pro- and anti-apoptotic proteins; it has been hypothesized that VDAC contributes to increase of mitochondrial permeability that is involved in the initiation of apoptosis.

We studied the effects of the mtDNA tRNA^Ile ^A4263G mutation on VDAC function and apoptosis. We report that expression level of VDAC mRNA in cell lines carrying the mutation was significantly decreased. Although the exact mechanism is unknown, it may be associated with dysfunction of energy metabolism and consequent increase in the levels of reactive oxygen species (ROS) [31]. We hypothesis that high apoptosis of the mutated cell lines was associated with increased expression of VDAC mRNA, which could be inhibited by the CsA, a kind of inhibitor of VDAC. In addition, imaging revealed that VDAC was co-localized with Bax protein in cell lines carrying the 4263 A→G mutation. These changes were accompanied by a decrease in the mitochondrial membrane potential ΔΨ_m _and increased levels of apoptosis versus control cell lines. The role of VDAC in these processes was confirmed by application of the selective VDAC inhibitor Cyclosporin A (CsA): the inhibitor abolished co-localization with Bax, restored ΔΨ_m _to levels of control CsA-treated cells, and decreased levels of apoptosis. Taken together, these results suggest that the mtDNA A4263G mutation, inferred to cause mis-charging of tRNA^Ile ^and consequent amino acid substitutions in mitochondrial proteins, may exert its pro-hypertensive effects by deregulating the expression of VDAC, in turn leading to increases in programmed cell death.

So we conclude that increased expression of mitochondrial VDAC and subcellular co-localization of VDAC/Bax increases mitochondrial permeability and apoptosis in cell lines carrying the mtDNA tRNA^Ile ^A4263G mutation.

Recent efforts to identify genes involved in essential hypertension (EHT) have focused on genetic markers and candidate genes in the nuclear genome. Nevertheless, the pathophysiological mechanisms underlying EHT remain unknown. Our results highlight the potential importance of mitochondrial genes in the etiology of hypertension. However, this work does not exclude a contribution from the nuclear genome, and maternal inheritance of a predisposition to EHT is likely to result from interactions between mtDNA and nuclear mutations. So further research will be required to elucidate the mechanisms whereby nuclear-mitochondrial interactions can predispose to the development of hypertension.

Finally this article we conclude that increased expression of mitochondrial VDAC and subcellular co-localization of VDAC/Bax increases mitochondrial permeability and apoptosis.

## Competing interests

The authors declare that they have no competing interests.

## Authors' contributions

Liu Yuqi, Gao Lei, Li Yang contributed equally to this article. Li Zongbin carried out the molecular genetic studies, participated in the sequence analysis and drafted the manuscript. Xu Hua and Wang Lin carried out the immunoassays. Chen Rui, Liu Mohan and Wen Yi participated in the cell line culture. Wang Shiwen and Guan Minxin conceived of the study, and participated in its design and coordination. All authors read and approved the final manuscript.

## Pre-publication history

The pre-publication history for this paper can be accessed here:

http://www.biomedcentral.com/1471-2350/10/114/prepub
